# Psychological distress and psychosocial care in adults with glaucoma in Africa: A systematic review protocol

**DOI:** 10.1371/journal.pone.0353202

**Published:** 2026-07-21

**Authors:** Chelsea Charlyne Kosoku, Albert Kwadjo Amoah Andoh, Constance Kukuaa Akuffo, Josephine Ampong, Nana Akwasi Owusu Mensah, Kwesi Sewe, Isaiah Osei Duah, Alexander K. Schuster, Simon Christoph König, Kwadwo Owusu Akuffo

**Affiliations:** 1 Department of Education and Psychology, College of Educational Studies, University of Cape Coast, Cape Coast, Ghana; 2 Department of Optometry and Visual Science, College of Science, Kwame Nkrumah University of Science and Technology, PMB, Kumasi, Ghana; 3 Department of Planning, College of Art and Built Environment, Kwame Nkrumah University of Science and Technology, PMB, Kumasi, Ghana; 4 University of Ghana Library System, University of Ghana, Legon-Accra, Ghana; 5 Department of Psychology, John R. and Kathy R. Hairston College of Health and Human Sciences, North Carolina Agricultural and Technical State University, Greensboro, North Carolina, United States of America; 6 Department of Ophthalmology, University Medical Centre Mainz, Centre for Ophthalmic Epidemiology and Healthcare Research, Mainz, Germany; University of Education Winneba Faculty of Science Education, GHANA

## Abstract

A diagnosis of glaucoma often carries a substantial psychological burden. The chronic nature of the disease, the prospect of lifelong treatment, the possibility of surgery, and concerns about progressive vision loss can contribute to significant psychological distress. Despite growing recognition of these emotional challenges, there has been no systematic synthesis of the burden of psychological distress among adults with glaucoma in Africa or of the psychological support services available within glaucoma care. This protocol, registered with the International Prospective Register of Systematic Reviews (PROSPERO: CRD420261338962), outlines a systematic review designed to address this gap. The review will comprehensively assess the prevalence and determinants of psychological distress among adults with glaucoma and identify interventions or support services integrated into glaucoma care across African settings. Searches will be conducted in PubMed, Scopus, CINAHL, Google Scholar, and African Journals Online without language or date restrictions. Retrieved records will be managed in EndNote and screened in Covidence by two independent reviewers, with disagreements resolved through consensus or consultation with a senior reviewer. Risk of bias and methodological quality will be evaluated using established critical appraisal tools. Where sufficient data are available, findings will be synthesized through meta-analysis; otherwise, a structured narrative synthesis following synthesis-without-meta-analysis principles will be undertaken. By consolidating the available evidence, this review aims to inform the integration of psychological care into glaucoma management and support more holistic approaches to preserving both visual and mental well-being.

## Introduction

Glaucoma is an insidious, progressive eye disease that damages the optic nerve and results in irreversible vision loss [1]. As a major cause of irreversible blindness, populations of African ancestry bear a disproportionate burden of primary open-angle glaucoma, the most common subtype, which is characterized by earlier onset, faster progression, and a higher risk of irreparable deterioration of vision [2,3]. Its asymptomatic early course, combined with high age-specific risk and the need for lifelong treatment, contributes to substantial psychological strain [4–7]. Beyond visual impairment, glaucoma is strongly associated with adverse mental health outcomes, including anxiety, depression, and stress, which are more prevalent in affected individuals compared with healthy controls [8–10]. This growing evidence has led to the recognition that psychological distress is an integral component of the glaucoma experience rather than merely a consequence of disease progression, underscoring the importance of integrating mental health care into routine ophthalmic management [11,12]. Failure to manage psychological distress may also jeopardize glaucoma prognosis and treatment outcomes [13,14].

Several theoretical frameworks help explain the psychological burden associated with glaucoma. For instance, Lazarus and Folkman’s transactional theory of stress and coping conceptualizes glaucoma as a chronic stressor that is appraised in terms of threat to autonomy, functional ability, and future independence, with perceived risk of vision loss triggering emotional distress and coping responses [15]. The Health Belief Model further suggests that distress and adherence behaviors are influenced by patients’ perceptions of disease severity, susceptibility to blindness, and barriers to treatment such as cost, medication burden, and access to care [16]. Complementing these, the Biopsychosocial Model emphasizes that psychological outcomes arise from the interaction of biological disease progression, individual psychological responses, and broader social conditions, including reduced functional capacity, loss of independence, and limited social support [17]. Together, these frameworks highlight that the impact of glaucoma extends beyond ocular pathology to encompass multidimensional determinants of mental health.

The psychological burden of glaucoma is further aggravated by limited social support and the demands of lifelong disease management, which are strongly associated with depression and anxiety [18]. Patients often experience persistent fear of blindness, reduced quality of life, sleep disturbances, and social withdrawal, all of which compound emotional distress and may adversely affect treatment adherence [19,20]. Epidemiological evidence across populations further suggests that approximately 40% of individuals with glaucoma experience anxiety and about 20% experience depression, rates significantly higher than those in the general population [21]. Despite the increasing recognition of these psychological outcomes, region-specific evidence from Africa remains limited, particularly regarding how vision loss interacts with psychosocial factors and the availability of supportive care systems in shaping mental health outcomes in this population [22].

### Rationale for this systematic review

Previous systematic reviews have consistently reported an association between glaucoma and psychological morbidity, including elevated levels of depression, anxiety, and sleep disturbance among affected individuals [21,23–26]. However, these syntheses are largely based on global evidence drawn predominantly from high- and middle-income settings, with a strong focus on prevalence estimates and clinical risk factors such as disease severity, age, and sex [21,23–26]. As a result, African populations remain substantially underrepresented despite bearing a disproportionate burden of glaucoma and related visual impairment. This limits the transferability of existing evidence to African health systems and leaves a critical gap in understanding how psychological distress manifests and is managed in this region.

Within African settings, prior studies have largely examined isolated patient groups rather than providing a continent-wide synthesis of mental health outcomes associated with glaucoma or the psychosocial care provided [20,28–32]. Existing reviews also pay limited attention to how mental health services are integrated into ophthalmic care pathways, particularly in resource-constrained settings [12,21,23,24,26,27]. Cultural beliefs about blindness, delayed presentation, limited mental health infrastructure, and barriers to continuity of care all shape both the experience of psychological distress and the feasibility of psychosocial support within routine glaucoma management [28–30].

No prior systematic review has specifically synthesized evidence on how psychological distress is assessed, reported, and managed within glaucoma care pathways in Africa. A focused systematic review is therefore required to consolidate available evidence, identify gaps in psychosocial care integration, and inform the development of multidisciplinary and patient-centered approaches tailored to African health systems. Psychiatric comorbidities may also interact with glaucoma outcomes and contribute to a cycle of physical decline and mental distress, further reinforcing the need for integrated care approaches [31]. This review seeks to address these gaps by synthesizing evidence on the prevalence, characteristics, and psychosocial care of psychological disorders associated with glaucoma in African settings, with the aim of informing strategies that improve adherence, quality of life, and overall disease outcomes.

## Materials and methods

**Fig 1:**
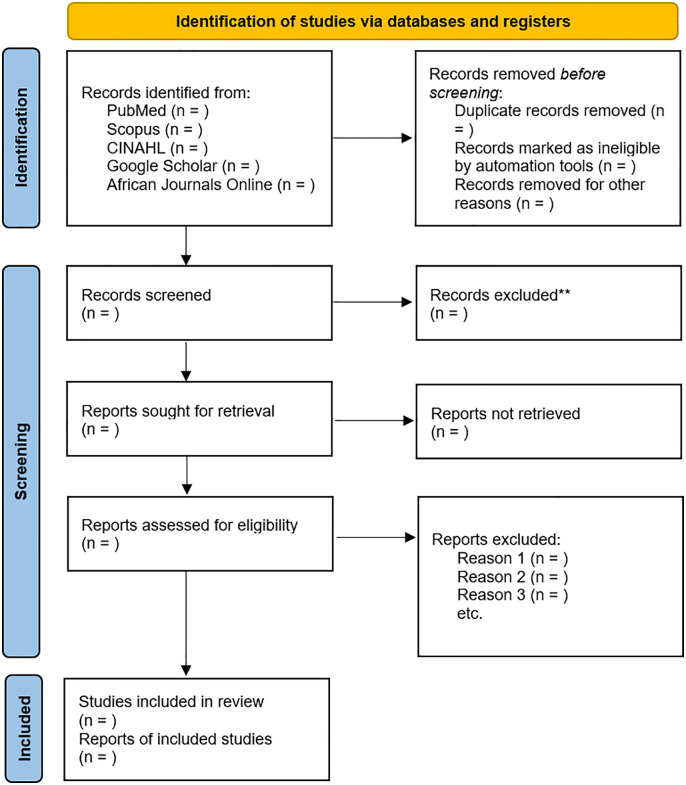
PRISMA flow diagram illustrating the study selection process.

The review protocol has been prepared in accordance with the Preferred Reporting Items for Systematic Reviews and Meta-Analysis Protocols (PRISMA-P) checklist (S1 Table) [32,33]. The protocol has also been indexed in the International Prospective Register of Systematic Reviews (PROSPERO ID: CRD420261338962), and the full review will be guided by the Preferred Reporting Items for Systematic Reviews and Meta-analysis (PRISMA) 2020 guidelines [34,35]. The PICOS framework will be adopted to describe the population, intervention, comparator, outcome, and study types. Study selection and database screening will be systematically documented using a PRISMA flow diagram, as seen in Fig 1.

### Criteria for including studies for this review

Eligibility criteria for this review will be defined according to the Population, Intervention, Comparator, Outcomes, and Study Design (PICOS) framework.

### Population

African adults (≥18 years) diagnosed with any type of glaucoma will be eligible for inclusion in this review. Caregivers of adults with glaucoma will also be eligible, provided that the psychological outcomes assessed are directly related to glaucoma diagnosis, management, or disease progression. The toolkit or criteria for diagnosing glaucoma (e.g., the International Society of Geographical and Epidemiological Ophthalmology criteria, etc.) and/or psychological distress such as (the Kessler Psychological Distress Scale – K6 or K10; General Health Questionnaire – GHQ12; Depression, Anxiety and Stress Scale – DASS 21/42; or the Patient Health Questionnaire - PHQ9/PHQ4, etc.)should have been reported. Persons with any form of psychological distress will be excluded if the study population consists exclusively of individuals diagnosed with other chronic progressive conditions aside from glaucoma, where glaucoma is not the primary condition of interest. Participants with comorbid chronic conditions within glaucoma cohorts will not be excluded. The study should include participants residing in any African country. Studies involving African nationals living outside Africa will be excluded.

### Intervention/exposure

Any psychological distress associated with glaucoma (clinically confirmed diagnosis) and/or psychosocial or mental health interventions integrated into glaucoma management, such as counseling services, psychoeducation, support groups, behavioral interventions, screening programs for psychological distress, or referral systems to mental health services. Studies evaluating routine glaucoma care without a psychological component will be excluded.

### Comparator

Where applicable, studies comparing psychological distress among individuals with glaucoma to persons without glaucoma, different stages or severities of glaucoma, or psychosocial intervention versus usual care groups will be included. However, the presence of a comparator group will not be mandatory for inclusion.

### Outcomes

Studies examining psychological distress outcomes among adults with glaucoma in African settings will be eligible irrespective of whether a psychosocial intervention was implemented. Primary outcomes: 1) prevalence and types of psychological distress among adults with glaucoma in Africa; 2) psychosocial or mental health care interventions incorporated into glaucoma management in African settings.

Secondary outcomes: 1) patient- and health system-related factors associated with psychological distress among adults with glaucoma in Africa.

### Study design

Eligible studies will include any cross-sectional, cohort, or case-control study that reports psychological outcomes among adults diagnosed with glaucoma in African countries. Intervention studies, qualitative studies, literature reviews, scoping reviews, and systematic reviews will not be included. However, the list of included/reviewed studies (especially from any retrieved global systematic review on the subject) will be perused for African studies for inclusion in this review. Case studies, case series (are atypical and do not represent the source population), opinions, commentaries, and editorial letters will be excluded.

### Data sources

The following electronic databases will be systematically searched from inception to 31st March 2026, without language restrictions: PubMed, Scopus, CINAHL (Cumulative Index to Nursing and Allied Health Literature), and Google Scholar. Google Scholar will be searched using simplified keyword combinations reflecting the core review concepts, as the platform has limited support for advanced Boolean operators, controlled vocabulary, and structured field searching. To ensure methodological feasibility, the first 200 results ranked by relevance will be screened. Additionally, the reference lists of all included studies and relevant review articles will be manually screened to identify further eligible studies. African Journals Online (AJOL) will be explored during preliminary searching but will not be retained as a primary database source due to limitations in search reproducibility, advanced search functionality, and record export capabilities, which may affect the transparency and replicability of the review process.

### Search terms and search strategies

A comprehensive search strategy will be developed using controlled vocabulary (e.g., MeSH terms) and free-text keywords. Search terms will include ‘glaucoma’, ‘psychological distress’, ‘depression’, ‘anxiety’, ‘psychosocial support systems’, ‘counseling’, and ‘mental health service’, fused with all applicable alternate terms and the names of African countries for running the search, as shown in Table 1. Search strategies will be adapted for each database and will be piloted and refined to maximize sensitivity. Full search strategies for each database are presented in data in S2 Appendix.

**Table 1:** Search strategy for PubMed.

Database	PICO Concept Block	Search Terms
PubMed	Population (Adults with Glaucoma)	(glaucoma*[tiab] OR “primary open angle glaucoma”[tiab] OR POAG[tiab] OR “angle closure glaucoma”[tiab] OR “secondary glaucoma”[tiab] OR Glaucoma[Mesh])
	Intervention/Exposure (Psychosocial Care)	“Psychosocial Support Systems”[Mesh] OR counseling[tiab] OR counselling[tiab] OR psychoeducation[tiab] OR psychotherapy[tiab] OR “psychological intervention*”[tiab] OR “mental health screening”[tiab] OR “support group*”[tiab] OR “psychosocial care”[tiab] OR “integrated care”[tiab] OR “collaborative care”[tiab] OR referral*[tiab] OR “service integration”[tiab] OR “mental health service*”[tiab] OR rehabilitation[tiab] OR “patient education”[tiab])
	Comparator	Not included in the search strategy, as comparator groups are not mandatory for inclusion.
	Outcomes (Psychological Distress)	(Depression[Mesh] OR Anxiety[Mesh] OR “Mental Health”[Mesh] OR “Stress, Psychological”[Mesh] OR depress*[tiab] OR anxiet*[tiab] OR “psychological distress”[tiab] OR “emotional distress”[tiab] OR stigma*[tiab] OR coping[tiab] OR “fear of blindness”[tiab] OR “quality of life”[Mesh])
	Setting (Africa)	(Africa[Mesh] OR “Africa South of the Sahara”[Mesh] OR Africa[tiab] OR “sub-Saharan Africa”[tiab] OR “Sub-Saharan African”[tiab] OR “North Africa”[tiab] OR “West Africa”[tiab] OR “East Africa”[tiab] OR “Central Africa”[tiab] OR “Southern Africa”[tiab] OR Algeria[tiab] OR Angola[tiab] OR Benin[tiab] OR Botswana[tiab] OR Burkina Faso[tiab] OR Burundi[tiab] OR “Cabo Verde”[tiab] OR “Cape Verde”[tiab] OR Cameroon[tiab] OR “Central African Republic”[tiab] OR Chad[tiab] OR Comoros[tiab] OR Congo[tiab]OR “Republic of the Congo”[tiab] OR “Democratic Republic of the Congo”[tiab] OR DRC[tiab] OR “Cote d’Ivoire”[tiab] OR “Côte d’Ivoire”[tiab] OR Ivory Coast[tiab] OR Djibouti[tiab] OR Egypt[tiab] OR “Equatorial Guinea”[tiab] OR Eritrea[tiab] OR Eswatini[tiab] OR Swaziland[tiab] OR Ethiopia[tiab] OR Gabon[tiab] OR Gambia[tiab] OR Ghana[tiab] OR Guinea[tiab] OR “Guinea-Bissau”[tiab] OR Kenya[tiab] OR Lesotho[tiab] OR Liberia[tiab] OR Libya[tiab] OR Madagascar[tiab] OR Malawi[tiab] OR Mali[tiab] OR Mauritania[tiab] OR Mauritius[tiab] OR Morocco[tiab] OR Mozambique[tiab] OR Namibia[tiab] OR Niger[tiab] OR Nigeria[tiab] OR Rwanda[tiab] OR “Sao Tome and Principe”[tiab] OR “São Tomé and Príncipe”[tiab] OR Senegal[tiab] OR Seychelles[tiab] OR “Sierra Leone”[tiab] OR Somalia[tiab] OR “South Africa”[tiab] OR “South Sudan”[tiab] OR Sudan[tiab] OR Tanzania[tiab] OR “United Republic of Tanzania”[tiab] OR Togo[tiab] OR Tunisia[tiab] OR Uganda[tiab] OR Zambia[tiab] OR Zimbabwe[tiab])

### Study selection

All retrieved citations will be exported into EndNote for reference management and duplicate removal. Subsequently, all references will be exported to Covidence for titles, abstract screening, and full text review. Screening will occur in two stages: 1) Title and Abstract Screening – where two independent reviewers (AKAA and JA) will screen titles and abstracts against eligibility criteria; 2) Full-Text Review – where potentially eligible studies will undergo full-text assessment by at least two reviewers (AKAA, JA, and CCK) independently. Disagreements at any stage will be resolved through discussion or consultation with a third reviewer (KOA). The study selection process will be documented using a PRISMA 2020 flow diagram shown in Fig 1. All studies excluded at the full-text stage will be listed, and the reason(s) for the exclusion will be provided.

### Data extraction

A standardized data extraction form will be developed and piloted prior to using randomly selected studies to ensure consistency between data extractors. At least two reviewers (AKAA, JA, and CCK) will independently extract data from included studies and transfer it to a Microsoft Excel spreadsheet. Study characteristics such as author(s), year of publication, country, study design, sample size and participant characteristics, type of glaucoma, psychological outcomes assessed, measurement tools used (e.g., PHQ-9/PHQ-4, K6/K10, GHQ-12, DASS-21/42, etc.), prevalence or severity of psychological distress, description of psychosocial interventions (if applicable), and barriers and facilitators to psychosocial integration will be extracted. For studies published in languages other than English, data will be extracted from full-text articles that have either been translated using language translation software or translated by bilingual team members with expertise in the subject. A second reviewer will verify the accuracy of the translations whenever possible, and any uncertainties will be documented and, if needed, clarified with the study authors. In studies where outcome data are missing or insufficiently reported, corresponding authors will be contacted via email to request the required information. In situations where it is not possible to obtain the missing data from the primary authors, the amount and reasons for the missing data will be reported. If data from multinational studies have been lumped together, we will attempt to disaggregate the data by country. Where it is impossible to do so, such studies will be presented as one, accompanied by citations of the included countries. Discrepancies in extracted data will be resolved through consensus.

### Risk of bias and quality assessment

The methodological quality of the included studies will be assessed using the Joanna Briggs Institute (JBI) critical appraisal checklists appropriate to each study design. Particularly, the JBI appraisal checklist designed for cross-sectional, cohort, and case-control studies will be applied where applicable for each included study design. Two reviewers (NAOM and JA) will independently assess each study for risk of bias across domains, including sampling methods, measurement validity and reliability, statistical analysis, and response rates. A determination of “low risk” of bias, “high risk” of bias, or “unclear” bias will be made for each bias domain. Discrepancies between reviewers will be resolved through discussion or consultation with a third reviewer (KOA) where necessary. Risk of bias and quality assessments will be summarized and reported in the final review.

### Data synthesis

Given the anticipated heterogeneity in study designs, psychological assessment instruments, outcome measures, and reporting approaches, findings will be synthesized narratively. Quantitative results will be summarized descriptively and organized according to key review domains, including population characteristics, the prevalence and types of psychological distress among individuals with glaucoma, psychosocial or mental health care components integrated into glaucoma management, and factors influencing the integration of psychosocial care within ophthalmic services. Studies will be grouped according to outcome domains, psychosocial care elements, and relevant contextual characteristics to facilitate comparison across settings and populations. Summary tables will be used to systematically present study characteristics and key findings. Where appropriate, evidence-mapping techniques will be employed to visually illustrate the distribution of evidence across countries, outcome domains, and psychosocial care components, thereby identifying patterns in the literature, evidence gaps, and priorities for future research. Narrative synthesis, supported by summary tables and evidence maps, will constitute the primary approach to evidence integration and interpretation.

### Confidence in cumulative evidence

The certainty of the evidence for each outcome will be assessed independently by two reviewers (JA and NAOM) using the Grading of Recommendations Assessment, Development, and Evaluation (GRADE) approach [36]. This framework evaluates evidence across five domains: risk of bias, inconsistency, indirectness, imprecision, and publication bias. Based on these criteria, the certainty of evidence will be rated as high, moderate, low, or very low. The GRADE assessment will be applied to contextualize the strength and reliability of conclusions drawn from the included studies.

### Handling of missing data

In cases where included studies report missing, incomplete, or unclear data, attempts will be made to contact the corresponding authors to obtain additional information or clarification, or where possible, ask for the raw data to enable us to extract the missing information. If the requested data cannot be retrieved, the extent and nature of the missing information will be documented and transparently reported.

### Study status and timeline

At the time of submission, the systematic review had not commenced data collection or analysis. Database searching, study screening, and data extraction will be undertaken in accordance with the pre-registered protocol on PROSPERO (ID: CRD420261338962). Data collection is planned to begin in April 2026 and is expected to be completed by June 2026. Data synthesis and manuscript preparation are anticipated to be finalized by August 2026. No results are currently available.

### Ethics and registration

As this review involves analysis of previously published studies, ethical approval is not required. Findings will be disseminated through peer-reviewed publications, conference presentations, and stakeholder engagement with ophthalmic and mental health practitioners in African settings.

### Patient and public involvement

Patients and members of the public were not  involved in the design of the review questions, outcomes to be assessed, or how the completed systematic review findings will be reported. Nonetheless, the review is designed with a patient-centered perspective, with a particular focus on the psychological impact of glaucoma and the integration of psychosocial support into routine ophthalmic care. The findings of this review will be relevant to patients and the wider public, and efforts will be made to disseminate results in formats that are accessible to both clinical and non-clinical audiences.

## Discussion

The chronic and irreversible nature of glaucoma, coupled with the threat of progressive vision loss, may generate sustained psychological strain among affected individuals [11,12]. Research indicates that the psychological burden of this condition is significantly influenced by a lack of perceived social support [18]. Understanding the psychological and psychosocial impact of living with glaucoma is particularly important in Africa, where individuals with visual impairment, especially those of lower socioeconomic status, experience higher rates of depression and anxiety [37,38], alongside a disproportionately high burden of glaucoma [39].

Furthermore, the global prevalence of glaucoma, particularly in Africa, emphasizes the urgency of developing culturally sensitive and economically viable interventions that extend beyond mere clinical management to encompass comprehensive psychosocial support [22,39–41]. Presently, psychological distress has been associated with poorer quality of life, reduced treatment adherence, and adverse psychosocial outcomes among individuals with glaucoma [22,42,43]. Therefore, the integration of psychological assessment and intervention within routine ophthalmological care becomes imperative for optimizing both mental health and clinical outcomes.

This review is expected to provide evidence that can directly inform the strengthening of glaucoma care pathways in African settings by identifying gaps in the systematic recognition and management of psychological distress within routine ophthalmic care. Specifically, the synthesis may highlight deficiencies such as the absence of routine mental health screening in glaucoma clinics, limited referral pathways between ophthalmology and mental health services, insufficient integration of counselling or psychosocial support into long-term glaucoma management, and variability in clinician awareness of the psychosocial burden of disease. More broadly, the review may inform patient-centered, multidisciplinary care frameworks that align glaucoma management with holistic health system approaches, particularly in resource-constrained African contexts where service fragmentation remains a key barrier to comprehensive chronic disease care. This holistic approach must integrate mental health care into ophthalmological practice, acknowledging the bidirectional relationship between psychological states and disease progression [44].

In summary, by bridging ophthalmology and mental health, the review aims to support the development of integrated care approaches that address not only visual outcomes but also the broader well-being of individuals living with glaucoma.

### Strengths and limitations

This systematic review has several notable strengths. It will provide the first comprehensive synthesis of evidence on psychological distress and psychosocial care in glaucoma, specifically within African populations, addressing a critical gap in geographically contextualized research. The use of a pre-registered protocol, proposed adherence to PRISMA guidelines, together with comprehensive multi-database search strategy without language restrictions will enhance methodological rigor, inclusivity, and reproducibility. However, some limitations are anticipated for this systematic synthesis of evidence. The expected heterogeneity in study designs, psychological assessment tools, and outcome reporting may limit comparability across studies and preclude quantitative synthesis. Restricting Google Scholar searches to the first 200 results may lead to the omission of relevant studies beyond this threshold. The exclusion of grey literature (e.g., theses, conference abstracts, and programme reports) may limit the inclusion of context-specific evidence, particularly from African settings where publication rates in peer-reviewed journals may be lower. Variability in the diagnostic criteria for glaucoma across included studies may introduce clinical heterogeneity, as studies may have used differing definitions and diagnostic standards depending on setting and resources. Furthermore, variations in methodological quality across included studies may influence the strength and interpretation of the overall evidence base.

### Implications of the anticipated review findings

The findings of this review are expected to have important implications for clinical practice, research, and health policy in African settings. By synthesizing evidence on the burden and nature of psychological distress among individuals with glaucoma, the review will underscore the need for integrating mental health assessment and support into routine ophthalmic care. Identification of gaps in how psychosocial care is incorporated into existing glaucoma management pathways will provide a foundation for developing contextually appropriate, multidisciplinary models of care that address both visual and psychological health. Furthermore, the review will inform future research priorities by highlighting deficiencies in the integration of psychosocial support within ophthalmic services and the need for structured, scalable approaches to mental health inclusion. At a policy level, the findings may support advocacy for embedding mental health services within eye care programs, ultimately contributing to improved treatment adherence, enhanced patient well-being, and better overall quality of life among individuals living with glaucoma in Africa.

## Supporting information

S1 Table PRISMA-P (Preferred reporting items for systematic review and meta-analysis protocols) 2015 checklist: Recommended items to address in a systematic review protocol. (DOCX)

S2 Appendix Full search strategy for each database. (DOCX)
