# Supplementary material for: Psychological distress and psychosocial care in adults with glaucoma in Africa: A systematic review protocol
Source: PLoS One. 2026 Jul 21;21(7):e0353202. doi: 10.1371/journal.pone.0353202 (PMC13387554; doi:10.1371/journal.pone.0353202)
Supplement: S2 Appendix — (DOCX) [file pone.0353202.s002.docx]

# Full Database Search Strategies

**1. PUBMED**

| **Database** | **PICO Concept Block** | **Search Terms** |
| --- | --- | --- |
| PubMed | Population (Adults with Glaucoma) | (glaucoma* OR "primary open angle glaucoma" OR POAG OR "angle closure glaucoma" OR "secondary glaucoma" OR Glaucoma) |
|  | Intervention/Exposure (Psychological Distress and Psychosocial Care) | ("psychological distress"OR "emotional distress" OR depress* OR anxiet* OR stigma* OR coping OR "fear of blindness" OR "mental health" OR "quality of life" OR Depression OR Anxiety OR "Mental Health" OR "Stress, Psychological" OR "Psychosocial Support Systems" OR counseling OR counselling OR psychoeducation OR psychotherapy OR "psychological intervention*" OR "mental health screening" OR "support group*" OR "psychosocial care" OR "integrated care" OR "collaborative care" OR referral* OR "service integration" OR "mental health service*" OR rehabilitation OR "patient education") |
|  | Comparator | Not included in the search strategy, as comparator groups are not mandatory for inclusion. |
|  | Outcomes (Psychological and Psychosocial Outcomes) | ("psychological distress" OR depress* OR anxiet* OR stress OR stigma* OR coping OR wellbeing OR "quality of life" OR "fear of blindness" OR Depression OR Anxiety OR "Quality of Life") |
|  | Setting (Africa) | (Africa OR "Africa South of the Sahara" OR Africa OR "sub-Saharan Africa" OR "Sub-Saharan African" OR "North Africa" OR "West Africa" OR "East Africa" OR "Central Africa" OR "Southern Africa" OR Algeria OR Angola OR Benin OR Botswana OR Burkina Faso OR Burundi OR "Cabo Verde" OR "Cape Verde" OR Cameroon OR "Central African Republic" OR Chad OR Comoros OR Congo OR "Republic of the Congo" OR "Democratic Republic of the Congo" OR DRC OR "Cote d'Ivoire" OR "Côte d'Ivoire" OR "Ivory Coast" OR Djibouti OR Egypt OR "Equatorial Guinea" OR Eritrea OR Eswatini OR Swaziland OR Ethiopia OR Gabon OR Gambia OR Ghana OR Guinea OR "Guinea-Bissau" OR Kenya OR Lesotho OR Liberia OR Libya OR Madagascar OR Malawi OR Mali OR Mauritania OR Mauritius OR Morocco OR Mozambique OR Namibia OR Niger OR Nigeria OR Rwanda OR "Sao Tome and Principe" OR "São Tomé and Príncipe" OR Senegal OR Seychelles OR "Sierra Leone" OR Somalia OR "South Africa" OR "South Sudan" OR Sudan OR Tanzania OR "United Republic of Tanzania" OR Togo OR Tunisia OR Uganda OR Zambia OR Zimbabwe |

**2. SCOPUS**

| **Database** | **PICO Concept Block** | **Search Terms** |
| --- | --- | --- |
| Scopus | Population (Adults with Glaucoma) | TITLE-ABS-KEY (glaucoma* OR "primary open angle glaucoma" OR POAG OR "angle closure glaucoma" OR "secondary glaucoma") |
|  | Intervention/Exposure (Psychological Distress and Psychosocial Care) | TITLE-ABS-KEY (depress* OR anxiet* OR "psychological distress" OR "emotional distress" OR stigma* OR coping OR wellbeing OR "fear of blindness" OR "quality of life" OR QoL OR HRQoL OR counsel* OR psychoeducation OR psycho-education OR psychotherapy OR "psychosocial support" OR "psychosocial intervention*" OR "support group*" OR "mental health screening" OR "mental health service*" OR "integrated care" OR "collaborative care" OR "service integration" OR referral* OR rehabilitation OR "patient education") |
|  | Comparator | Comparator terms were not included in the search strategy because comparator groups are not mandatory for study inclusion. |
|  | Outcomes (Psychological and Psychosocial Outcomes) | TITLE-ABS-KEY (depress* OR anxiet* OR "psychological distress" OR stress OR stigma* OR coping OR wellbeing OR "fear of blindness" OR "quality of life" OR QoL OR HRQoL OR "mental health") |
|  | Setting (Africa) | TITLE-ABS-KEY (africa* OR "sub saharan africa" OR "West Africa" OR "North Africa" OR "East Africa" OR "Central Africa" OR "Southern Africa" OR algeria OR angola OR benin OR botswana OR "burkina faso" OR burundi OR "cape verde" OR "cabo verde" OR cameroon OR chad OR "central african republic" OR comoros OR congo OR "democratic republic of congo" OR djibouti OR egypt OR eritrea OR eswatini OR swaziland OR ethiopia OR gabon OR gambia OR ghana OR guinea OR "guinea bissau" OR "cote d'ivoire" OR "ivory coast" OR kenya OR lesotho OR liberia OR libya OR madagascar OR malawi OR mali OR mauritania OR mauritius OR morocco OR mozambique OR namibia OR niger OR nigeria OR rwanda OR senegal OR seychelles OR "sierra leone" OR somalia OR "south africa" OR "south sudan" OR sudan OR tanzania OR togo OR tunisia OR uganda OR zambia OR zimbabwe) |

**3. CINAHL**

| **Database** | **PICO Concept Block** | **Search Terms** |
| --- | --- | --- |
| CINAHL | Population (Adults with Glaucoma) | ((MH "Glaucoma+") OR (TI glaucoma* OR AB glaucoma*) OR "primary open angle glaucoma" OR POAG OR "angle closure glaucoma" OR "secondary glaucoma") |
|  | Intervention/Exposure (Psychological Distress and Psychosocial Care) | ((MH "Depression+") OR (MH "Anxiety+") OR (MH "Mental Health+") OR (MH "Psychological Stress+") OR depress* OR anxiet* OR "psychological distress" OR "emotional distress" OR stigma* OR coping OR wellbeing OR "fear of blindness" OR "quality of life" OR QoL OR HRQoL OR (MH "Psychosocial Support Systems") OR counseling OR counselling OR psychoeducation OR psychotherapy OR "psychosocial intervention*" OR "support group*" OR "mental health screening" OR "mental health service*" OR "integrated care" OR "collaborative care" OR referral* OR rehabilitation OR "patient education") |
|  | Comparator | Comparator terms were not included in the search strategy because comparator groups are not mandatory for study inclusion. |
|  | Outcomes (Psychological and Psychosocial Outcomes) | ((MH "Depression+") OR (MH "Anxiety+") OR (MH "Mental Health+") OR depress* OR anxiet* OR "psychological distress" OR stress OR stigma* OR coping OR wellbeing OR "fear of blindness" OR "quality of life" OR QoL OR HRQoL) |
|  | Setting (Africa) | ((MH "Africa+") OR Africa OR "sub-Saharan Africa" OR "West Africa" OR "North Africa" OR "East Africa" OR "Central Africa" OR "Southern Africa" OR Algeria OR Angola OR Benin OR Botswana OR "Burkina Faso" OR Burundi OR Cameroon OR Chad OR Congo OR Egypt OR Ethiopia OR Ghana OR Kenya OR Malawi OR Morocco OR Mozambique OR Namibia OR Nigeria OR Rwanda OR Senegal OR "South Africa" OR Sudan OR Tanzania OR Tunisia OR Uganda OR Zambia OR Zimbabwe) |

**4. AJOL**

glaucoma depression
glaucoma anxiety
glaucoma "psychological distress"
glaucoma counseling

**5. Google Scholar (Publish Or Perish searches)**

glaucoma depression Africa
glaucoma anxiety Africa
glaucoma "psychological distress" Africa
glaucoma counseling Africa
